# Investigating the effectiveness of three school based interventions for preventing psychotic experiences over a year period – a secondary data analysis study of a randomized control trial

**DOI:** 10.1186/s12889-023-15107-x

**Published:** 2023-02-01

**Authors:** Lorna Staines, Colm Healy, Paul Corcoran, Helen Keeley, Helen Coughlan, Elaine McMahon, Padraig Cotter, David Cotter, Ian Kelleher, Camilla Wasserman, Romuald Brunner, Michael Kaess, Marco Sarchiapone, Christina W. Hoven, Vladimir Carli, Danuta Wasserman, Mary Cannon

**Affiliations:** 1 Department of Psychiatry, Royal College of Surgeons in Ireland, 123 St Stephens Green, Dublin, Ireland; 2 National Suicide Research Foundation, Cork, Ireland; 3 School of Public Health, University College Cork, Cork, Ireland; 4 Child and Adolescent Mental Health Services North Cork, Health Service Executive, Cork, Ireland; 5 Research Society of Process Oriented Psychology United Kingdom (RSPOPUK), Old Hampstead Townhall 213 Haverstock Hill, NW3 4QP London, UK; 6 Park Royal Centre for Mental Health, Central and North West London (CNWL) NHS Trust, Central Way, Off Acton Lane, NW10 7NS London, UK; 7 Department of Psychiatry, Beaumont Hospital, Dublin 9, Ireland; 8 Division of Psychiatry, Centre for Clinical Brain Sciences, University of Edinburgh, EH10 5HF Edinburgh, UK; 9 Department of Child and Adolescent Psychiatry, Columbia University, New York State Psychiatric Institute, New York, NY USA; 10 National Centre for Suicide Research and Prevention of Mental lll-Health (NASP), Karolinska Institute, Stockholm, Sweden; 11 Clinic for Child and Adolescent Psychiatry, Psychosomatics and Psychotherapy, University of Regensburg, Regensburg, Germany; 12 University Hospital of Child and Adolescent Psychiatry and Psychotherapy, University of Bern, Bern, Switzerland; 13 Department of Child and Adolescent Psychiatry, Center for Psychosocial Medicine, University Hospital Heidelberg, Heidelberg, Germany; 14 Department of Medicine and Health Science, University of Molise, Campobasso, Italy; 15 Department of Epidemiology, Mailman School of Public Health, Columbia University, New York, USA

**Keywords:** Intervention, Psychotic experiences, School based intervention, Prevention, Psychosis

## Abstract

**Introduction:**

Psychotic experiences (PEs) are associated with increased risk of later mental disorders and so could be valuable in prevention studies. However, to date few intervention studies have examined PEs. Given this lack of evidence, in the current study a secondary data analysis was conducted on a clustered-randomized control trial (RCT) of 3 school based interventions to reduce suicidal behaviour, to investigate if these may reduce rates of PEs, and prevent PE, at 3-month and 1-year follow-up.

**Methods:**

The Irish site of the Saving and Empowering Young Lives in Europe study, trial registration (DRKS00000214), a cluster-RCT designed to examine the effect of school-based interventions on suicidal thoughts and behaviour. Seventeen schools (n = 1096) were randomly assigned to one of three intervention arms or a control arm. The interventions included a teacher training (gate-keeper) intervention, an interactive educational (universal-education) intervention, and a screening and integrated referral (selective-indicative) intervention. The primary outcome of this secondary data-analysis was reduction in point-prevalence of PEs at 12 months. A second analysis excluding those with PEs at baseline was conducted to examine prevention of PEs. Additional analysis was conducted of change in depression and anxiety scores (comparing those with/without PEs) in each arm of the intervention. Statistical analyses were conducted using mixed-effects modelling.

**Results:**

At 12-months, the screening and referral intervention was associated with a significant reduction in PEs (OR:0.12,95%CI[0.02–0.62]) compared to the control arm. The teacher training and education intervention did not show this effect. Prevention was also observed only in the screening and referral arm (OR:0.30,95%CI[0.09–0.97]). Participants with PEs showed higher levels of depression and anxiety symptoms, compared to those without, and different responses to the screening and referral intervention & universal-education intervention.

**Conclusions:**

This study provides the first evidence for a school based intervention that reduce & prevent PEs in adolescence. This intervention is a combination of a school-based screening for psychopathology and subsequent referral intervention significantly reduced PEs in adolescents. Although further research is needed, our findings point to the effectiveness of school-based programmes for prevention of future mental health problems.

**Supplementary Information:**

The online version contains supplementary material available at 10.1186/s12889-023-15107-x.

## Background

Prevention is key to public mental health just as it is key to public health generally [1]. Prevention for mental health focuses on two key issues: Reducing severe psychopathology prior to developing mental disorder, and preventing new incidence of psychopathology [2–4]. Identifying and reducing early risk markers would be a valuable route to implementing mental health prevention. One such risk factor are psychotic experiences (PEs). PEs are hallucinations/delusions which can occur outside of a psychotic disorder and in the general population [5]. PEs are generally transitory and remit [6], and are considered as an early indicator of developing mental ill health [7]. PEs are common within the general population (~ 5%)[8], particularly in youth (between 8–17%)[9]. PEs are associated with a 4-fold increased risk for psychotic disorder, and a 3-fold increased risk for any mental disorder [10]. PEs are also associated with suicidal thoughts and behaviours [11–13] poorer functioning [14–16], healthcare needs [17–19] and psychiatric multi-morbidity [9, 20]. Adverse outcomes have been found even in those who report transient PEs [10, 15, 16, 19, 21, 22]. PEs and psychopathology are significantly associated [23, 24] and show a bi-directional relationship [25]. This substantial association has been proposed as evidence that PEs represent a marker of severe psychopathology [26, 27].

In comparison to intervention research, prevention is likely to be a more effective approach for treatment, due to the difficulties in recovery from a disorder [28–30], and additional deficits in functioning following a disorder [31, 32]. Prevention of PEs could be a valuable avenue of inquiry, particularly in the context of PEs and psychopathology. Additionally, studies have found participants who report PEs in addition to mental disorders, show slower rates of recovery when in treatment, due to higher rates of symptomology at baseline [33, 34]. Current school based prevention interventions often don’t differentiate between those with/without ill-health at baseline [4], which can achieve prevention aim of reducing psychopathology [2, 3], but does not determine if interventions can stop new incidence of psychopathology [4].

To date few studies have focused specifically on interventions for subclinical psychotic symptoms [35], and a majority which do exist rely on a clinical high risk model [35–37], which may represent only a small proportion of psychotic disorder [38–40]. Interventions for PEs without these criteria are rare [41–44]. Only one study to date has had a large (n > 1000) sample size [43], and did find that digital cognitive behavioural therapy was effective. One study to date has focused on PEs prevention [45], finding resilience training was effective at reducing PEs in a college sample (n = 107). To date, one intervention [42], and no prevention studies, have examined these approaches in adolescence, despite the highest incidence of PEs being in adolescence [46]. Additionally, to our knowledge, no study has examined the effectiveness of school-based interventions for preventing PEs.

Given the lack of knowledge regarding the efficacy of preventing PEs using school based interventions, we opted to conduct a secondary data analysis on a pre-existing school based randomized control trial (RCT) examining a suicide ideation & behaviour intervention. The aim of our study is to investigate the potential effectiveness of three school-based interventions for preventing PEs over a one-year period. We examined prevention in two ways (1) Reduction, at a whole group level were there less PEs? & (2) Prevention, were there fewer incident PEs? Our secondary aim was to examine whether there was a change in depression and anxiety scores following these interventions in those who reported PEs at baseline.

## Methods

### Ethical considerations

Ethical approval for the Saving and Empowering Young Lives in Europe (SEYLE) study was sought at each site of the study. For the Irish site, this was granted by the Clinical Research Ethics Committee of the Cork Teaching Hospitals. Three arms of the study (Control, Question-Persuade-Refer & Youth Aware of Mental Health) had opt-out consent and the other (Professional Screening) had opt-in consent. For safety during the RCT, students across all treatment arms who indicated acute suicide risk during baseline or follow-up assessments were immediately contacted by the SEYLE team and invited for a subsequent interview and referral to care.

### Trial design

Full details for the SEYLE study have been described elsewhere [47, 48]. Briefly, SEYLE study is a clustered-RCT, investigating the effectiveness of prevention strategies for suicidal behaviour in adolescents across 11 countries. The study was registered at the German Clinical Trials Registry (DRKS00000214). A full breakdown of the methodology, implementation, and cost effectiveness of the SEYLE study can be found in [47–50]. Full CONSORT checklist are available (eTable 1).

### Study setting

The Irish site was the only site that incorporated questions on PEs into the study questionnaire [51], and so was the only included site for this study. The Irish site identified twenty-four schools in the south-west of Ireland who were approached for participation. Of these, 17 participated. Informed consent and assent was obtained from a parent/guardian and all participating students. For three arms (Control, Question-Persuade-Refer & Youth Aware of Mental Health) flyers were sent home and opt-out consent was used. One arm (Screening by Professional and Refer) had opt-in consent, and so required parental signature to participate.

### Participants

**Fig. 1:**
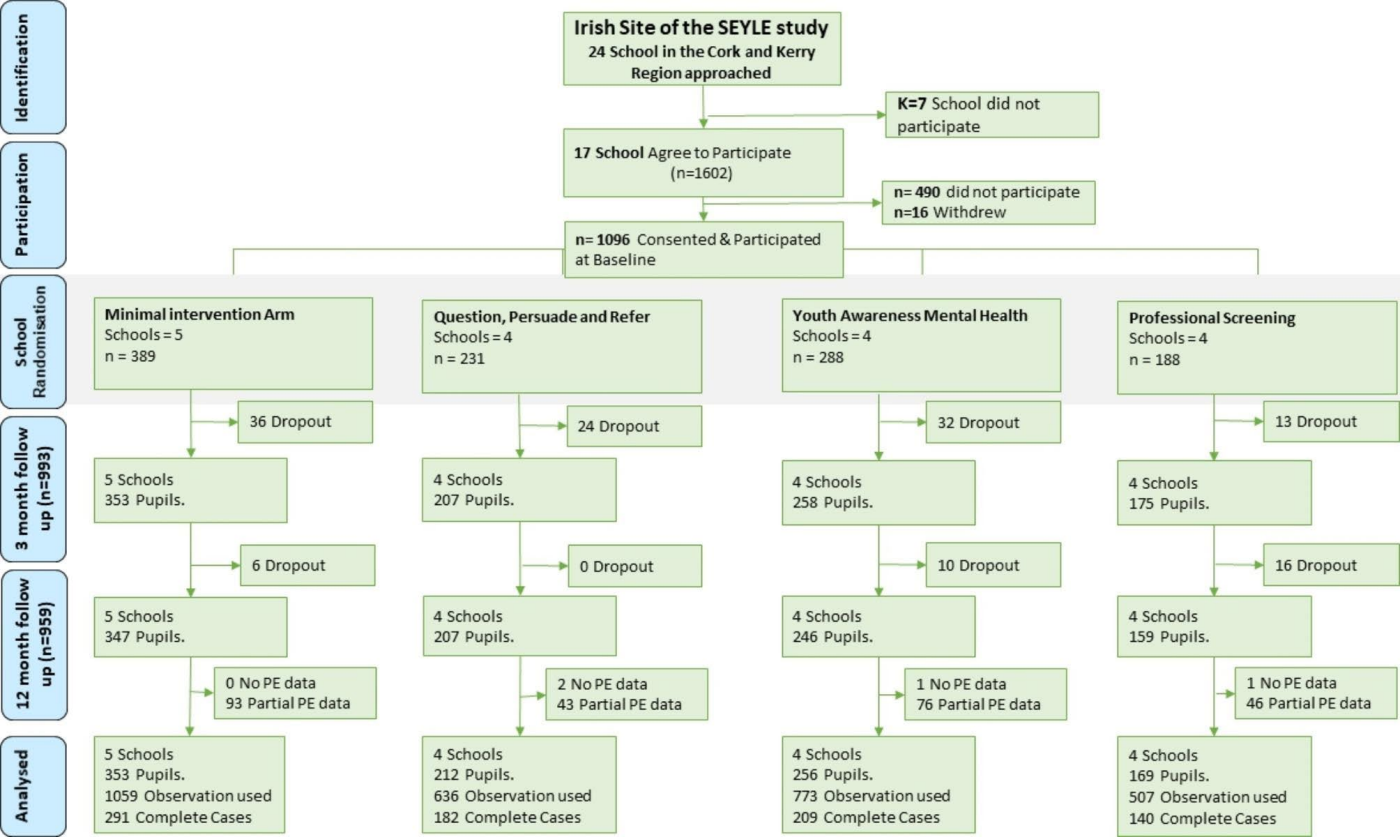
CONSORT diagram of the Irish site of the SEYLE study. (Note: Grey shading indicates the intervention period. Partial PEs data indicates the number of individuals who provided at least one wave of PEs data. These individuals were included in the main analysis. A supplementary analysis restricted to participants who provided data at all three waves of the study is presented in eAnalysis 1)

Participants were school-students, aged 13–15 years old. Students in classes where the majority were 14 years old were invited to participate (n = 1602) and 69% (n = 1112) completed a baseline self-report questionnaires. Follow-up questionnaires were administered at 3-months and 12-months after baseline, with 89% (n = 993) and 86% (n = 959) taking part at 3-months and 12-months, respectively (Fig. 1). For additional participant information (see eMethods).

### Interventions

The SEYLE study consisted of three active intervention arms and one control arm: (1) A gatekeeper intervention: Question, Persuade and Refer (QPR), (2) A psychoeducational intervention: Youth Aware of Mental health programme (YAM), (3) A universal screener and selective intervention: Screening by Professional and Refer (ProfScreen) and (4) A Minimal Intervention arm (Control). The interventions were provided between baseline and the three-month follow-up. Two interventions (QPR, YAM) use a universal intervention approach, while ProfScreen utilizes a universal screening and selective-indicative intervention approach.

A full description of each arm can be found in [48].

1. The QPR is a standardised gate-keeper programme [52]. For the SEYLE study, QPR was used to train teachers and school staff to recognise the risk of suicidal behaviour in students and improve their communication skills with students to motivate the at-risk pupil to seek professional help.
2. The YAM is a standardised mental health awareness programme developed for the SEYLE study targeting pupils [53]. This intervention includes interactive lectures on mental health, a series of role-play sessions and workshops, a 32-page information booklet that pupils could take home and psycho-educational posters that were hung in the classrooms.
3. ProfScreen is a two-stage school-based screening approach, which was developed for the SEYLE study[54]. In the first step, students exceeding one or more predetermined cut-offs (eTable 2) on psychopathology, risk-behaviour, or both, on the baseline screening questionnaire were considered “at-risk”. Endorsement of PEs was not a criterion for ProfScreen referral. In cases where a participant was determined as “at-risk”, the child’s parents were contacted by phone and letter to arrange an interview with a mental health professional.

The ProfScreen clinical interview was a semi-structured clinical interview, which was based on the Schedule for Affective Disorders and Schizophrenia for School-Age Children [55], and carried out by a child and adolescent psychiatrist or registrar trainee. It was developed to assess the need for mental healthcare, rather than to determine a psychiatric diagnosis. The interview was used to distinguish between pupils with psychological problems that required referral to mental healthcare and those who did not. Wherever possible, cut-offs were defined according to DSM-IV diagnostic criteria. All those who were considered in need were referred for appropriate intervention. Interviews were arranged between the baseline assessment and 3-month follow-up.

4) The Control arm were, for ethical reasons, provided with minimal intervention. Psycho-educational posters were displayed in the pupils’ classrooms. Contact information for local health care providers was provided on these posters.

### Outcomes

Measurement of Psychotic Experiences: PEs were measured at baseline, three-month follow-up and 12-month follow-up using a question on auditory hallucinations from the Adolescent Psychotic Symptom Screener (APSS)[51]. This question has been shown to have excellent psychometric properties [56] and a “definite” endorsement of this items has been validated against clinical interview with excellent sensitivity and specificity for all PEs [51]. This was the only question from the APSS that was included at all waves of the study.

Measurement of Depression: The Beck Depression Inventory II (BDI) was used as a self-reported measure of depression at all waves of the study[57]. The BDI is a 21-item clinically validated screening tool for depression with higher scoring indicating greater levels of depression.

Measurement of Anxiety: The Zung Self-Rating Anxiety Scale (SAS) was used as a self-reported measure of anxiety at all waves of the study[58]. The SAS is a 20-item clinically validated screening tool for anxiety with higher scoring indicating greater levels of anxiety.

Measurement of demographics: Information was obtained at baseline assessment on the participant’s age, gender, and nationality (Irish or non-Irish). In addition, participants were asked to respond “yes” or “no” to the question “Have you, during the past 12-months, been physically attacked?”. Physical victimization is used a measure of bullying, known to be a confounder to PE rates [59].

### Sample size

The target sample for each intervention arm as well as for the control arm is 250 pupils, i.e. 1,000 subjects in each participating country [48]. For the present study, a total of 1096 were included from the Irish site, including all four arms; Controls (n = 353), QPR (n = 212), YAM (n = 256), ProfScreen (n = 169). Previous prevention studies for PE [45] had a sample of 107, and Cohen’s d of 0.57, utilizing this power, we estimate the need for a minimum of 102 per group to accurately calculate the research questions for the current study.

### Randomization

Randomization was done at a school level, 17 schools which consented to participated were randomized into one of the arms of the study. Schools were stratified into large (> median school size in Ireland) or small (< median school size in Ireland) and randomized using a random number generator [48].

### Statistical Methods

All analysis was completed using Stata 14 [60].

#### Aim 1

A mixed-effect logit model with random effect of school was used, due to intervention clustering, and accounting for the within-subject repeated-measures effect. The main effects of intervention arm, time and their interactions are reported. Interaction effects (examining the effectiveness of the intervention) at 3-months and 12-months are displayed in odds ratios at 3-months and 12-months are displayed in odds ratios before and after adjustment-1 (baseline psychotic experiences) and adjustment-2 (baseline psychotic experiences, age, gender, nationality and exposure to physical victimisation). A stratified analysis excluded those who reported PEs at baseline, to examine prevention of PEs. Secondary data analysis were conducted on only complete cases using the same statistical technique (eAnalysis 1).

#### Aim 2

Depression and anxiety symptom scores were grouped by intervention arm, and further divided by PE status at baseline. The main effects, the interaction between those with/without PEs at baseline, and time, on depression and anxiety scores, were examined. Depression scores formed a negative binomial distribution and were analysed using a mixed effect negative binomial model. The anxiety scores were normally distributed, thus we applied a linear mixed effects model.

## Results

### Baseline Demographics and Clinical Characteristics

**Table 1:** Baseline demographic and clinical profiles of study, based on intervention arm

	Total	Control	QPR	YAM	ProfScreen
Demographic Characteristic					
Age	13.7(0.7)	13.6(0.5)	13.7(0.5)	13.7(0.6)	**14.2**(1.0)*
Gender, % male	54.7	54.5	46.8	64.2*	50.5
Nationality, % Irish	81.9	86.3	76.3*	75.4*	89.7
Clinical Characteristics					
AH %(n)	7.1(76)	5.3(20)	6.1(14)	8.1 (23)	**10.4**(19)*
Physical Victimization %(n)	10.0(109)	10.0(39)	5.6(13)	13.5(39)	9.57(18)
Zung Anxiety Score m(SD)	31.6(7.5)	31.2(7.3)	31.3(7.4)	31.4(7.3)	**33.2**(8.4)*
Becks Depression Index score m (SD)	6.8(7.3)	6.7(6.9)	6.3(6.9)	6.9(7.4)	7.5(8.3)

Note * = p < .05. Controls were the reference category in all comparisons.

Participants in the ProfScreen group were slightly older than controls (β = 0.56, 95%CI: 0.45–0.68, p < .001)) (Table 1). There was a higher percentage of males in the YAM group relative to controls (χ^2^ = 6.47, p = .01). The QPR(χ^2^ = 15.12, p < .001) and YAM (χ^2^ = 13.22, p < .001) had slightly lower percentage of Irish born participants relative to controls (Table 1). A greater percentage of participants assigned to the ProfScreen arm reported PEs at baseline than the control arm (RR:2.07,CI:1.08–3.99,p = .029). They also had higher anxiety scores at baseline (β:1.95,CI:0.56–3.35,p = .006) but this effect size was small (η^2^ = 0.01,CI:0.001–0.041).

### Participants with and without PEs

In the total sample and within each study arm, there were no significant differences in age, gender or nationality between participants with and without PEs (eTable 3). There were significant differences in physical victimisation, depression and/or anxiety scores between those with and without PEs in the overall sample and within the study arms (eTable 3).

#### Aim 1

Examining the effect of the interventions on the point prevalence of PEs at follow-up.

Over the course of the study 11% (n = 120) of study participants reported PEs on at least one occasion. PEs were reported by 7.1% (n = 76) of participants at baseline, 5.3% (n = 5) at 3-months and 4.5% (n = 42) at 12-months.

**Table 2:** Effect sizes for each intervention arm at 3 months and 12 months on point prevalence & incidence of PEs.

		Univariate		Adjustment 1 Baseline PEs		Adjustment 2 Baseline PEs & other characteristics*		Incidence Removal of baseline PEs
		OR (95%CI)		OR (95%CI)		OR (95%CI)		OR (95%CI)
QPR	3-month	0.83 (0.19–3.65)		0.79 (0.18–3.48)		0.75 (0.16–3.45)		1.06 (0.31–3.67)
	12-month	0.30 (0.07–1.43)		0.37 (0.08–1.65)		0.39 (0.08–1.83)		0.32 (0.10–1.07)
YAM	3-month	1.26 (0.35–4.47)		1.25 (0.36–4.37)		1.30 (0.37–4.56)		1.06 (0.33–3.39)
	12-month	0.53 (0.14-2.00)		0.63 (0.17–2.30)		0.75 (0.20–2.75)		0.57 (0.18–1.79)
ProfScreen	3-month	0.71 (0.18–2.83)		0.69 (0.18–2.76)		0.67 (0.16–2.72)		1.62 (0.49–5.35)
	12-month	**0.11** (0.02–0.58)		**0.14** (0.03–0.68)		**0.12** (0.02–0.62)		0.30* (0.09–0.97)

* = Other characteristics include age, gender, nationality and physical victimisation.

**Fig. 2:**
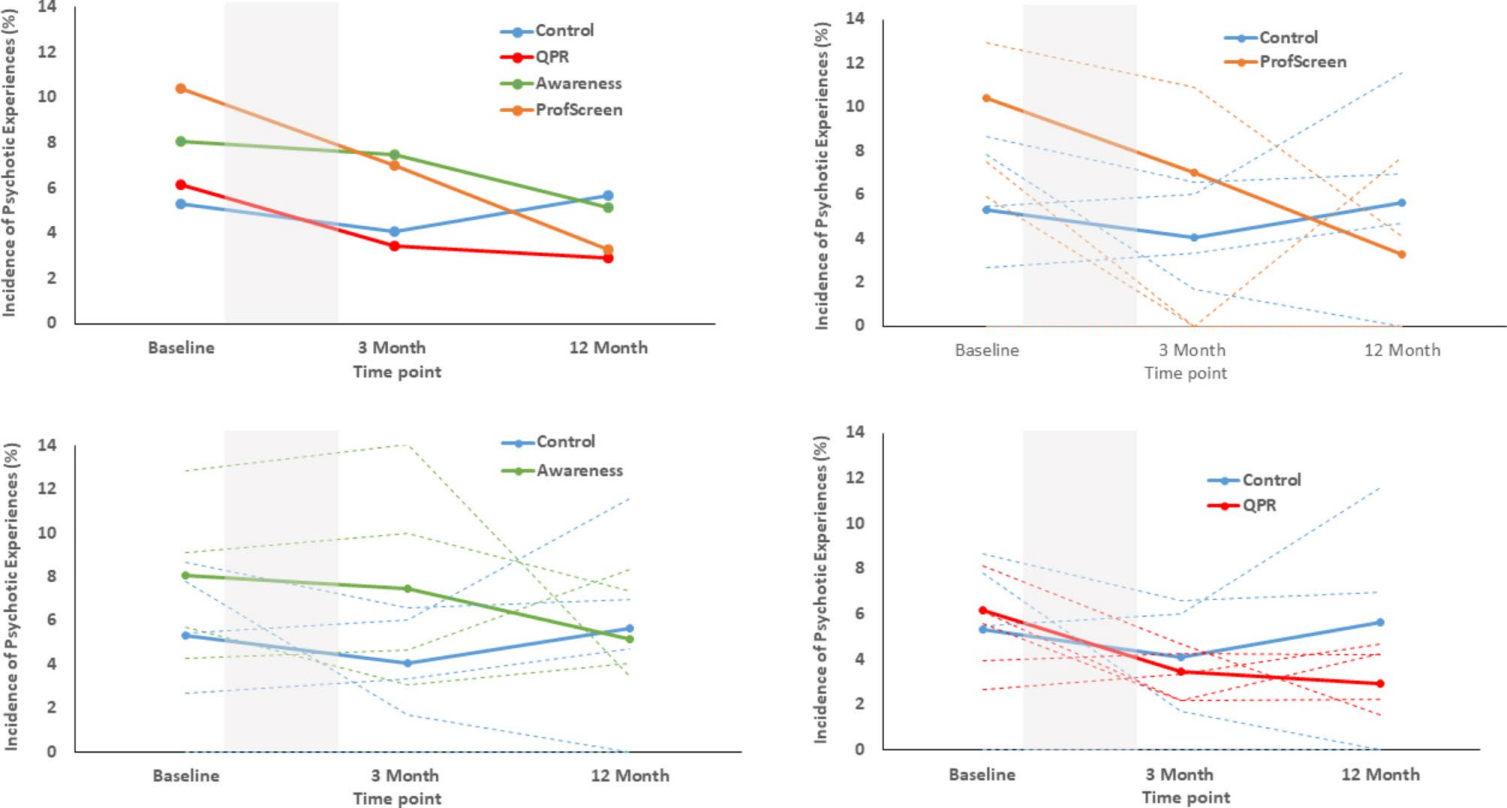
The point prevalence of psychotic experiences at baseline, 3-months and 12-months follow-up in each arm of the study. (Note: Dotted line is the point prevalence in each of the participating schools. Grey shading indicates the intervention period)

There was no significant main effect of intervention arm, time or interaction between intervention arm and time at 3-months follow-up. At 12-months follow-up, there was a significant interaction indicating a reduction in the point prevalence of PEs in the ProfScreen arm when compared to the Control arm (Fig. 2). This effect was retained after adjustment (Table 2). A test for trend indicated a significant linear effect in the ProfScreen arm over the three time points (χ^2^ = 11.42,p < .001). There were no other significant interactions, or linear or quadratic effects in any group.

Excluding those who reported PEs at baseline, there was no significant main effect at 3-month for any intervention arm(Table 2). At 12-month, there was a significantly lower incidence of PE in the ProfScreen arm, compared to controls (Table 2). No other arm showed a significantly lower number of PEs, compared to controls (Table 2). Secondary data analysis (eAnalysis 1) of complete cases and excluding those who reported PEs at baseline showed a similar reduction in PEs only in the ProfScreen arm at 12-months.

#### Aim 2

To examine whether there are changes in depression & anxiety scores in those with and without PEs at baseline.

#### Depression Scores

**Fig. 3:**
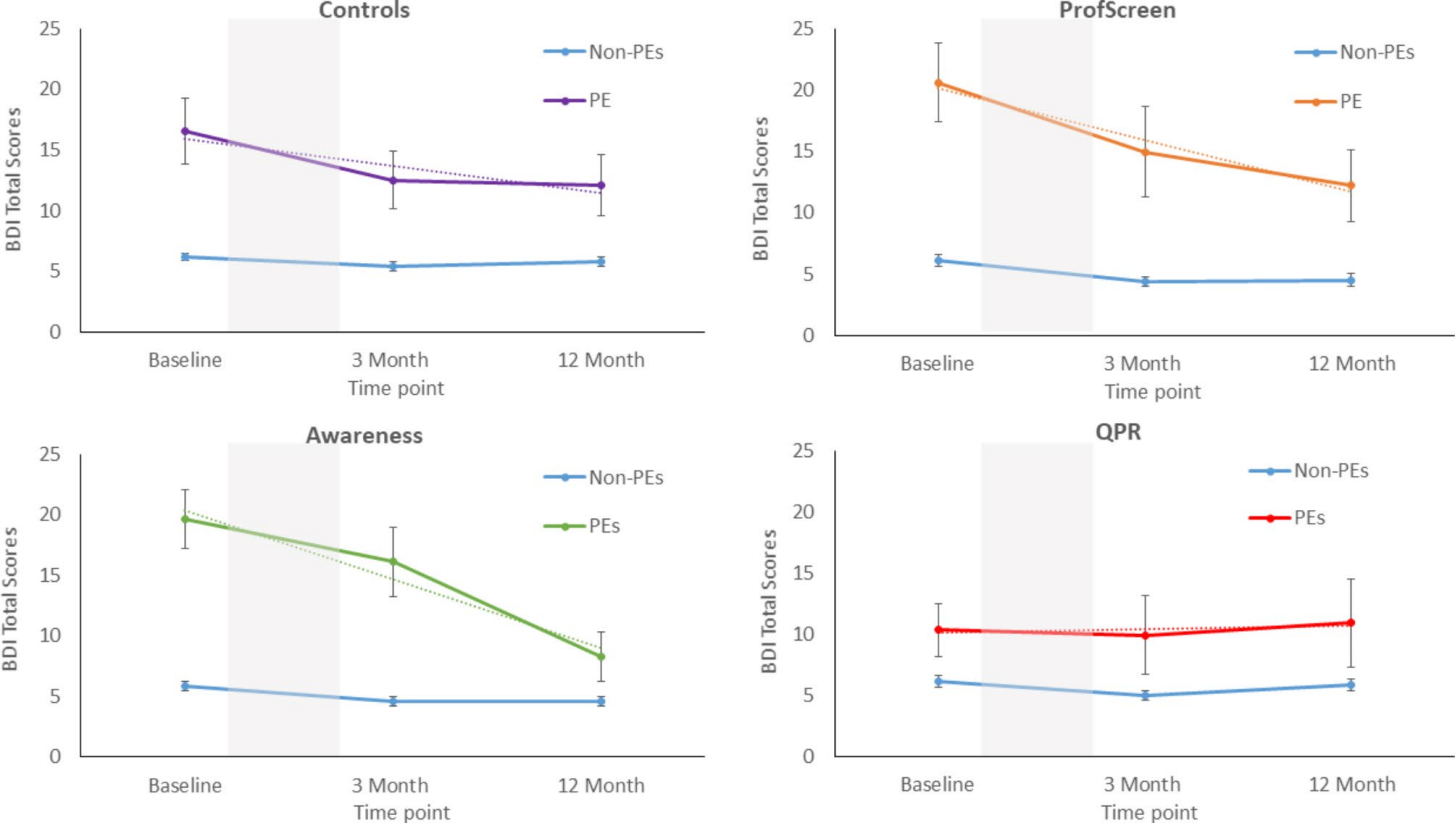
Depression scores at baseline, 3-months and 12-months follow-up in each arm of the study when stratified by the presence or absence of psychotic experiences at baseline. (Note: Beck depression Inventory. Grey shading indicates the intervention period. Dotted lines are linear trend lines. Error bars represent +/-1 standard error of the mean)

There were significant main effects of PE group and time in all arms of the study. This indicated that participants with baseline PEs had significantly higher depression scores than their peers and that depression scores were lower at follow-up relative to baseline (Fig. 3). We observed an interaction between PEs group and time in the ProfScreen arm (3-months:IRR:0.67,CI:0.57–0.79,p < .001; 12-months:IRR:0.63,CI:0.39–1.01,p = .057) and in the YAM arm at 12-months (3-months:IRR:1.05,CI:0.91–1.21,p = .477; 12-months:IRR:0.59,CI:0.40–0.85,p = .005). Both suggested that those with baseline PEs had a greater reduction in depression scores relative to those without baseline PEs. There was no interaction in either the control or the QPR arms (eFigure 3). Adjusting for baseline depression and other characteristics had very little effect on the interaction effect size in the ProfScreen arm (3-months:IRR:0.70,CI:0.69–0.73,p < .001; 12-months:IRR:0.71,CI:0.49–1.02,p = .06) (eFigure 1). However, there was some attenuation in the interaction in the YAM arm (3-months:IRR:0.97,CI:0.79–1.19,p = .80 12-months:IRR:0.62,CI:0.36–1.07,p = .08) (eFigure 2).

#### Anxiety Scores

**Fig. 4:**
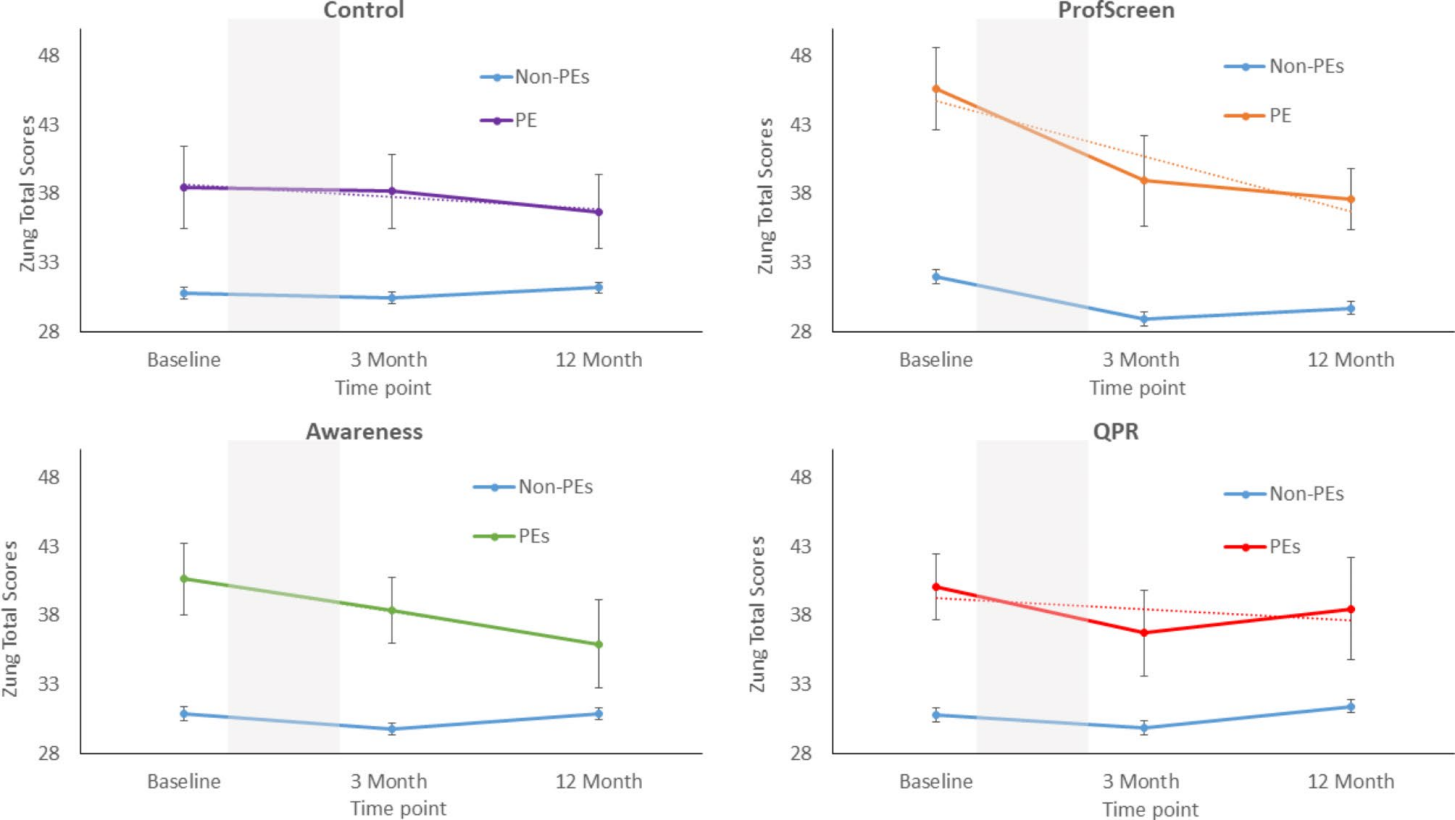
Anxiety scores at baseline, 3-months and 12-months follow-up in each arm of the study when stratified by the presence or absence of psychotic experiences at baseline. (Note: Zung Anxiety Score. Grey shading indicates the intervention period. Dotted lines are linear trend lines. Error bars represent +/-1 standard error of the mean)

There were significant main effects of PEs group in all study arms, which indicated that participants with baseline PEs had significantly higher anxiety scores than their peers (Fig. 4). We observed an interaction between PEs group and time in the ProfScreen arm at 3-months (β:-4.76,CI:-5.61- -3.92,p < .001) and 12-months (β:-4.20,CI:-5.59- -2.81,p = .001), indicating a greater reduction in anxiety scores in those with PEs when compared with their peers (Fig. 4). There was also an interaction between PEs and time on anxiety scores in the control arm at 12-months (β:-4.63,CI:-8.52- -0.74,p = .02). There was no interaction in either of the other two intervention arms (eFigs. 2 and 3). All significant interactions were retained even after adjustment for baseline anxiety scores and other characteristics (eFigure 1).

## Discussion

There is growing interest in a prevention based approach to mental disorders [61, 62]. This study aimed to examine for the first time the effectiveness of a school-based intervention to reduce and prevent PEs. We utilized a pre-existing RCT for suicide prevention to conduct a secondary data analysis, therefore results should be viewed as indicative of potential efficacy of school based interventions. Within this context, we found; The universal psychopathology screener, and integrated referral intervention (ProfScreen) showed a significant reduction and prevention in 12-month point prevalence of PEs, compared to controls. The universal & gate keeper intervention arms (YAM and QPR), did not show an effect on 12-month reduction or incidence of PEs. Those with PEs reported higher depression & anxiety scores than those without PEs at baseline. In the ProfScreen arm, those with PEs showed significantly greater reductions in both depression and anxiety scores, than their peers without baseline PEs.

Our results suggest that a two-stage screening and referral system can be effective at reducing the point prevalence and incidence of PEs at 12-months. Our results expand the findings from the university setting [43, 45] by highlighting that certain school-based interventions can reduce and prevent PEs. Potential explanations for this improvement may be due to the direct effect of the intervention with a health professional. Previous research on the SEYLE study found those who received the ProfScreen intervention did show a general (but non-significant) trend of higher help-seeking behaviour than controls [63]. However, previous research [64] examining those who were referred from the ProfScreen intervention, found overall only 38% of the participants in the SEYLE study attended the ProfScreen intervention. In the Irish site, 37% attended, and ranges across sites was substantial, from 5.7% in Italy, to 96.7% in France [64] .

The reason for the efficacy of the universal screener and selective intervention in this study then may be better explained by the act of being contacted by a health professional, rather than the meeting with the professional. Previous RCTs have observed an improved effect of intervention driven by more frequent follow-up contact [65]. For SEYLE the phone calls, letter, and/or referral, may have resulted in greater family awareness of potential problems. Previous research has shown parental support and lower child-parent conflict mediate the relationship between childhood adversity and PEs [66], and PEs and subsequent psychopathology [67]. It is also possible that the monitoring of psychiatric symptoms and the awareness that professional help was actively available assisted in reducing psychiatric morbidity, as has been observed in clinical studies [68]. Finally, it is possible that families sought supports/services outside of the ProfScreen intervention, although data is not available to examine service use of non-attendant referral participants for the SEYLE study.

An alternative explanation may be a down-stream effect of interventions. The primary goal of the SEYLE study was to examine the effectiveness of interventions for suicidal behaviour, rather than PEs. PEs are associated with suicidal thoughts and behaviour [12]. Another study has shown that CBT for improving sleeping patterns has been shown to reduce PEs in university students [43]. Evidence appears to show that interventions designed for different primary outcomes can reduce rates of PEs. Therefore, interventions which target symptoms known to be associated with PEs, can also improve PEs, without being a primary target i.e. a down-stream effect. The mechanisms for the ProfScreen intervention effect require further investigation.

Neither the teacher training (QPR) or universal psychoeducational (YAM) intervention showed significant evidence of preventing PEs. The universal psychoeducational intervention did reduce depression scores in young people with PEs at 12-months. In the full SEYLE sample, this intervention also significantly reduced the incidence of suicidal ideation & attempts at 12-month follow-up [47]. The differing outcomes of the current study may be as the interventions were insufficiently specialized for PEs i.e. the YAM arm was universal but with a focus on depression and suicidality. This interpretation is supported by the effectiveness of the universal screener and referral intervention arm (ProfScreen), which screened for psychopathology, highly associated with PEs [27, 69], and was not only targeting suicidality, but general poor mental health. However, specifically designed universal school-based interventions for PEs would need to be examined before such a result could be concluded.

Our secondary aim found those who reported PEs at baseline showed a greater improvement in anxiety and depression scores, relative to those without PEs, following the ProfScreen intervention. Previous research has observed differences in outcomes for PEs with and without co-occurring mental disorders [19, 69], and a recent intervention study found those with PEs showed slower rates of recovery from anxiety and depressive disorders [33]. In line with this research [33], those who reported baseline PEs showed substantially higher depression and anxiety symptoms, relative to those without PEs. This may therefore support literature of PEs being a marker of more severe psychopathology [27, 38, 69]. Within this context, studies which aim at prevention of PEs may be valuable to improving mental wellness.

### Limitations

The main limitation of the study was that the primary goal of the SEYLE study was to examine the effectiveness of interventions for suicidal behaviour, rather than PEs. Only the Irish sites used PEs as a measure, limiting the numbers involved. Additionally, due to the random sampling design, more individuals with PEs at baseline happened to be in the schools which were assigned to the ProfScreen arm. This was controlled for in analysis, but with a larger sample of schools i.e. the whole SEYLE sample, this random imbalance was unlikely to have occurred. Finally, PEs were measured using a single item (auditory hallucinations). Auditory hallucinations been shown have good clinical and construct validity for measuring PEs [51]. However, future studies should broaden their PEs definitions to include items on other psychotic phenomena.

Another consideration is a selective intervention approach can lead to stigma [70]. Selectively targeting students may result in peer scrutiny, which could increase isolation. We propose that perhaps the most effective preventative treatment may be a selective interventions, embedded within a universal intervention. It is plausible that this combination would provide psycho-educational information for all young people while simultaneously identifying those most at-risk. Such an intervention requires investigation of the efficacy and cost.

### Implication and future directions

Prevention and reduction is an important area of PE research; Firstly, PEs, even when transient, show long-term poorer functioning, psychopathology, and elevated healthcare needs [10, 15, 16, 19, 21, 22]. Therefore PEs prevention could be a valuable in improving general well-being. School based prevention interventions efficacy has been questioned, a recent systematic review of school-based prevention studies concluded that there was little evidence of their effectiveness for reducing symptoms of anxiety and depression [71]. The conclusions of Caldwell et al., [71] have been challenged, with [4] identifying, among other limitations, that examining reduction, but not incidence, is not truly reflective of prevention. Building from this proposal, our study examined all intervention arms for reduction and prevention. In line with Fazel et al., [4], and subsequent work [72, 73], our study supports the role of school-based preventions, finding positive improvements in PEs.

This study utilized pre-existing data to examine preliminary utility of school-based interventions in PEs prevention. There is now a need to replicate and expand these findings using a specifically designed RCT. However, based on these results, a priority should be given to approaches incorporating a universal screening tool, and referral interventions.

## Electronic supplementary material

Below is the link to the electronic supplementary material.

Supplementary Material 1

## Data Availability

The data are not publicly available due to privacy and ethical concerns. Specifically, the data used in this study has a human sample, and collected sensitive information. In this context, the study did not seek participant ethical approval for public release of data. Additionally, LS the corresponding author is a data processor, not a data controller of the SEYLE data and so cannot provide data access upon request. Prof Wasserman is the data controller, and access must be sought from them for data use.

## Declarations

Three arms of the study (Control, Question-Persuade-Refer & Youth Aware of Mental Health) had opt-out consent and the other (Professional Screening) had opt-in consent. Informed consent and assent was obtained from a parent/guardian and all participating students.

## Abbreviations

PEs: Psychotic experiences
CBT: Cognitive behavioural trial
RCT: Randomized control trial
SEYLE: Saving and Empowering Lives in Europe
QPR: Question, Persuade, Refer
YAM: Youth Aware of Mental Health
ProfScreen: Professional Screening

## References

[1] Campion J, Javed A, Vaishnav M, Marmot M (2020). Public mental health and associated opportunities. Indian J Psychiatry.

[2] Fusar-Poli P, Correll CU, Arango C, Berk M, Patel V, Ioannidis JPA. Preventive psychiatry: a blueprint for improving the mental health of young people. World Psychiatry. 2021 Jun;20(2):200–21. 10.1002/wps.20869 PMC8129854 34002494

[3] Cowen EL (2000). Now that we all know that primary prevention in mental health is great, what is it?. J Community Psychol.

[4] Fazel M, Kohrt BA. Prevention versus intervention in school mental health.Lancet Psychiatry. 2019 Dec1;6(12):969–71. 10.1016/S2215-0366(19)30440-7 31734107

[5] Staines L, Healy C, Coughlan H, Clarke M, Kelleher I, Cotter D, et al. Psychotic experiences in the general population, a review; definition, risk factors, outcomes and interventions. Psychol Med. 2022 Aug;25:1–12. 10.1017/S0033291722002550 PMC9772919 36004805

[6] McGorry P, Nelson B. Why We Need a Transdiagnostic Staging Approach to Emerging Psychopathology, Early Diagnosis, and Treatment. JAMA Psychiatry. 2016 Mar 1;73(3):191–2. 10.1001/jamapsychiatry.2015.2868 26765254

[7] McGorry PD, Hartmann JA, Spooner R, Nelson B (2018). Beyond the “at risk mental state” concept: transitioning to transdiagnostic psychiatry. World Psychiatry.

[8] van Os J, Linscott RJ, Myin-Germeys I, Delespaul P, Krabbendam L. A systematic review and meta-analysis of the psychosis continuum: evidence for a psychosis proneness–persistence–impairment model of psychotic disorder. Psychol Med. 2009 Feb;39(2):179–95. 10.1017/S0033291708003814 18606047

[9] Kelleher I, Connor D, Clarke MC, Devlin N, Harley M, Cannon M. Prevalence of psychotic symptoms in childhood and adolescence: a systematic review and meta-analysis of population-based studies. Psychol Med. 2012 Sep;42(9):1857–63. 10.1017/S0033291711002960 22225730

[10] Healy C, Brannigan R, Dooley N, Coughlan H, Clarke M, Kelleher I, et al. Childhood and adolescent psychotic experiences and risk of mental disorder: a systematic review and meta-analysis. Psychol Med. 2019 Jul;49(10):1589–99. 10.1017/S0033291719000485 31088578

[11] Honings S, Drukker M, Groen R, van Os J. Psychotic experiences and risk of self-injurious behaviour in the general population: a systematic review and meta-analysis. Psychol Med. 2016 Jan;46(2):237–51. 10.1017/S0033291715001841 26419206

[12] Yates K, Lång U, Cederlöf M, Boland F, Taylor P, Cannon M et al. Association of Psychotic Experiences With Subsequent Risk of Suicidal Ideation, Suicide Attempts, and Suicide Deaths: A Systematic Review and Meta-analysis of Longitudinal Population Studies. JAMA Psychiatry. 2019 Feb 1;76(2):180–9. 10.1001/jamapsychiatry.2018.3514 PMC6439738 30484818

[13] Hielscher E, DeVylder JE, Saha S, Connell M, Scott JG. Why are psychotic experiences associated with self-injurious thoughts and behaviours? A systematic review and critical appraisal of potential confounding and mediating factors. Psychol Med. 2018 Jul;48(9):1410–26. 10.1017/S0033291717002677 28929996

[14] Calkins ME, Moore TM, Satterthwaite TD, Wolf DH, Turetsky BI, Roalf DR, et al. Persistence of psychosis spectrum symptoms in the Philadelphia Neurodevelopmental Cohort: a prospective two-year follow‐up. World Psychiatry. 2017 Feb;16(1):62–76. 10.1002/wps.20386 PMC5269480 28127907

[15] Carey E, Gillan D, Healy C, Dooley N, Campbell D, McGrane J et al. Early adult mental health, functional and neuropsychological outcomes of young people who have reported psychotic experiences: a 10-year longitudinal study.Psychol Med. 2020;1–9. 10.1017/S0033291720000616 32216843

[16] Healy C, Campbell D, Coughlan H, Clarke M, Kelleher I, Cannon M (2018). Childhood psychotic experiences are associated with poorer global functioning throughout adolescence and into early adulthood. Acta Psychiatr Scand.

[17] Rimvall MK, van Os J, Verhulst F, Wolf RT, Larsen JT, Clemmensen L, et al. Mental Health Service Use and Psychopharmacological Treatment following psychotic Experiences in Preadolescence. Am J Psychiatry. 2020 Feb;26(4):318–26. 10.1176/appi.ajp.2019.19070724 32098486

[18] Rimvall MK, Wolf RT, Olsen EM, Skovgaard AM, Clemmensen L, Oxholm AS et al. Healthcare Costs, School Performance, and Health-related Quality of Life in Adolescence Following Psychotic Experiences in Preadolescence: A Longitudinal Cohort Study. Schizophr Bull [Internet]. 2020 Dec 21 [cited 2021 Mar 1];(sbaa175). Available from: 10.1093/schbul/sbaa175 10.1093/schbul/sbaa175 PMC8673435 33345286

[19] Bhavsar V, Dorrington S, Morgan C, Hatch SL, McGuire P, Fusar-Poli P, et al. Psychotic experiences, psychiatric comorbidity and mental health need in the general population: a cross-sectional and cohort study in Southeast London. Psychol Med. 2021 Jan;51(1):147–57. 10.1017/S0033291719003106 PMC7116680 31713511

[20] Kelleher I, Devlin N, Wigman JTW, Kehoe A, Murtagh A, Fitzpatrick C, et al. Psychotic experiences in a mental health clinic sample: implications for suicidality, multimorbidity and functioning. Psychol Med. 2014 Jun;44(8):1615–24. 10.1017/S0033291713002122 24025687

[21] Bromet EJ, Nock MK, Saha S, Lim CCW, Aguilar-Gaxiola S, Al-Hamzawi A, et al. Association between psychotic experiences and subsequent suicidal thoughts and behaviors: a cross-national analysis from the World Health Organization World Mental Health surveys. JAMA Psychiatry. 2017 Nov;74(1):1136–44. 10.1001/jamapsychiatry.2017.2647 PMC5710219 28854302

[22] Trotta A, Arseneault L, Caspi A, Moffitt TE, Danese A, Pariante C et al. Mental Health and Functional Outcomes in Young Adulthood of Children With Psychotic Symptoms: A Longitudinal Cohort Study.Schizophr Bull. 2020 Feb26;46(2):261–71. 10.1093/schbul/sbz069 PMC7442396 31361314

[23] Lancefield KS, Raudino A, Downs JM, Laurens KR (2016). Trajectories of childhood internalizing and externalizing psychopathology and psychotic-like experiences in adolescence: a prospective population-based cohort study. Dev Psychopathol.

[24] Healy C, Gordon AA, Coughlan H, Clarke M, Kelleher I, Cannon M (2019). Do childhood psychotic experiences improve the prediction of adolescent psychopathology? A longitudinal population-based study. Early Interv Psychiatry.

[25] McGrath JJ, Saha S, Al-Hamzawi A, Andrade L, Benjet C, Bromet EJ, et al. The bidirectional Associations between psychotic Experiences and DSM-IV Mental Disorders. Am J Psychiatry. 2016 Mar;17(10):997–1006. 10.1176/appi.ajp.2016.15101293 PMC5175400 26988628

[26] van Os J, Guloksuz S. A critique of the ‘ultra-high risk’ and ‘transition’ paradigm. World Psychiatry Off J World Psychiatr Assoc WPA. 2017 Jun;16(2):200–6. 10.1002/wps.20423 PMC5428198 28498576

[27] Stochl J, Khandaker GM, Lewis G, Perez J, Goodyer IM, Zammit S, et al. Mood, anxiety and psychotic phenomena measure a common psychopathological factor. Psychol Med. 2015 May;45(7):1483–93. 10.1017/S003329171400261X 25394403

[28] Huxley P, Krayer A, Poole R, Prendergast L, Aryal S, Warner R. Schizophrenia outcomes in the 21st century: A systematic review.Brain Behav. 2021 May15;11(6):e02172. 10.1002/brb3.2172 PMC8213926 33991072

[29] Jääskeläinen E, Juola P, Hirvonen N, McGrath JJ, Saha S, Isohanni M, et al. A systematic review and Meta-analysis of recovery in Schizophrenia. Schizophr Bull. 2013 Nov;39(6):1296–306. 10.1093/schbul/sbs130 PMC3796077 23172003

[30] Whiteford HA, Harris MG, McKeon G, Baxter A, Pennell C, Barendregt JJ, et al. Estimating remission from untreated major depression: a systematic review and meta-analysis. Psychol Med. 2013 Aug;43(8):1569–85. 10.1017/S0033291712001717 22883473

[31] Copeland WE, Alaie I, Jonsson U, Shanahan L. Associations of Childhood and Adolescent Depression with Adult Psychiatric and Functional Outcomes. J Am Acad Child Adolesc Psychiatry. 2021 May;60(1):604–11. 10.1016/j.jaac.2020.07.895 PMC8051642 32758528

[32] Degnan A, Berry K, Sweet D, Abel K, Crossley N, Edge D. Social networks and symptomatic and functional outcomes in schizophrenia: a systematic review and meta-analysis. Soc Psychiatry Psychiatr Epidemiol. 2018 Sep 1;53(9):873–88. 10.1007/s00127-018-1552-8 PMC6133157 29951929

[33] Knight C, Russo D, Stochl J, Croudace T, Fowler D, Grey N et al. Prevalence of and recovery from common mental disorder including psychotic experiences in the UK Primary Care Improving Access to Psychological Therapies (IAPT) Programme.J Affect Disord. 2020 Jul1;272:84–90. 10.1016/j.jad.2020.04.015 32379625

[34] Knight C, Russo D, Stochl J, Jones PB, Perez J. More sensitive identification of psychotic experiences in common mental disorder by primary mental healthcare services – effect on prevalence and recovery: casting the net wider. BJPsych Open [Internet]. 2020 Nov [cited 2021 Nov 8];6(6). Available from: https://www.cambridge.org/core/journals/bjpsych-open/article/more-sensitive-identification-of-psychotic-experiences-in-common-mental-disorder-by-primary-mental-healthcare-services-effect-on-prevalence-and-recovery-casting-the-net-wider/503A57B9628A03D5ADE7AB1238810C37 10.1192/bjo.2020.120 PMC7745243 33153513

[35] Soneson E, Russo D, Stochl J, Heslin M, Galante J, Knight C, et al. Psychological interventions for people with psychotic experiences: a systematic review and meta-analysis of controlled and uncontrolled effectiveness and economic studies. Aust N Z J Psychiatry. 2020 Jul;54(7):673–95. 10.1177/0004867420913118 PMC7324911 32462893

[36] Yung AR, Yung AR, Pan Yuen H, Mcgorry PD, Phillips LJ, Kelly D et al. Mapping the Onset of Psychosis: The Comprehensive Assessment of At-Risk Mental States.Aust N Z J Psychiatry. 2005 Nov1;39(11–12):964–71. 10.1080/j.1440-1614.2005.01714.x 16343296

[37] Miller TJ, McGlashan TH, Rosen JL, Cadenhead K, Ventura J, McFarlane W (2003). Prodromal Assessment with the structured interview for Prodromal Syndromes and the scale of prodromal symptoms: predictive validity, interrater reliability, and training to reliability. Schizophr Bull.

[38] Van Os J, Guloksuz S (2017). A critique of the “ultra-high risk” and “transition” paradigm. World Psychiatry.

[39] Lång U, Yates K, Leacy FP, Clarke MC, McNicholas F, Cannon M (2022). Systematic review and Meta-analysis: psychosis risk in children and adolescents with an At-Risk Mental State. J Am Acad Child Adolesc Psychiatry.

[40] Cotter D, Healy C, Staines L, Mongan D, Cannon M. Broadening the Parameters of Clinical High Risk for Psychosis. Am J Psychiatry [Internet]. 2022 Sep [cited 2022 Sep 12];179(9). Available from: 10.1176/appi.ajp.20220612 10.1176/appi.ajp.20220612 36048484

[41] Maddox L, Jolley S, Laurens KR, Hirsch C, Hodgins S, Browning S, et al. Cognitive behavioural therapy for unusual experiences in children: a case series. Behav Cogn Psychother. 2013 May;41(3):344–58. 10.1017/S1352465812000343 22874646

[42] Maijer K, Staring T, Bartels-Velthuis AA, Palmen SJ, Sommer IE. Stronger than your voices: A cognitive behavioral therapy for youth suffering from auditory verbal hallucinations: Clin Child Psychol Psychiatry [Internet]. 2019 Nov 21 [cited 2022 May 26]; Available from: https://journals.sagepub.com/doi/full/10.1177/1359104519888011 10.1177/1359104519888011 31749371

[43] Freeman D, Sheaves B, Goodwin GM, Yu LM, Nickless A, Harrison PJ et al. The effects of improving sleep on mental health (OASIS): a randomised controlled trial with mediation analysis.Lancet Psychiatry. 2017 Oct 1;4(10):749–58. 10.1016/S2215-0366(17)30328-0 PMC5614772 28888927

[44] Langer ÁI, Cangas AJ, Gallego J. Mindfulness-based intervention on Distressing Hallucination-Like Experiences in a nonclinical sample. Behav Change. 2010 Sep;27(3):176–83.

[45] DeTore NR, Luther L, Deng W, Zimmerman J, Leathem L, Burke AS et al. Efficacy of a transdiagnostic, prevention-focused program for at-risk young adults: a waitlist-controlled trial. Psychol Med. 2022 Mar 1;1–10. 10.1017/S0033291722000046 PMC9433469 35227342

[46] Sullivan SA, Kounali D, Cannon M, David AS, Fletcher PC, Holmans P, et al. A Population-Based Cohort Study examining the incidence and impact of psychotic Experiences from Childhood to Adulthood, and prediction of psychotic disorder. Am J Psychiatry. 2020 Jan;7(4):308–17. 10.1176/appi.ajp.2019.19060654 31906710

[47] Wasserman D, Hoven CW, Wasserman C, Wall M, Eisenberg R, Hadlaczky G et al. School-based suicide prevention programmes: the SEYLE cluster-randomised, controlled trial. The Lancet. 2015 Apr 18;385(9977):1536–44. 10.1016/S0140-6736(14)61213-7 25579833

[48] Wasserman D, Carli V, Wasserman C, Apter A, Balazs J, Bobes J et al. Saving and Empowering Young Lives in Europe (SEYLE): a randomized controlled trial.BMC Public Health. 2010 Apr13;10(1):192. 10.1186/1471-2458-10-192 PMC2880291 20388196

[49] Ahern S, Burke LA, McElroy B, Corcoran P, McMahon EM, Keeley H, et al. A cost-effectiveness analysis of school-based suicide prevention programmes. Eur Child Adolesc Psychiatry. 2018 Oct;27(10):1295–304. 10.1007/s00787-018-1120-5 29442231

[50] Carli V, Wasserman C, Wasserman D, Sarchiapone M, Apter A, Balazs J, et al. The saving and empowering young lives in Europe (SEYLE) Randomized Controlled Trial (RCT): methodological issues and participant characteristics. BMC Public Health. 2013 May;16:13:479. 10.1186/1471-2458-13-479 PMC3665603 23679917

[51] Kelleher I, Harley M, Murtagh A, Cannon M. Are Screening Instruments Valid for Psychotic-Like Experiences? A Validation Study of Screening Questions for Psychotic-Like Experiences Using In-Depth Clinical Interview. Schizophr Bull. 2011 Mar 1;37(2):362–9. 10.1093/schbul/sbp057 PMC3044617 19542527

[52] Tompkins TL, Witt J, Abraibesh N. Does a gatekeeper suicide prevention program work in a school setting? Evaluating training outcome and moderators of effectiveness. Suicide Life Threat Behav. 2010 Oct;40(5):506–15. 10.1521/suli.2010.40.5.506 21034213

[53] Wasserman C, Hoven CW, Wasserman D, Carli V, Sarchiapone M, Al-Halabí S et al. Suicide prevention for youth–a mental health awareness program: lessons learned from the Saving and Empowering Young Lives in Europe (SEYLE) intervention study. BMC Public Health. 2012 Sep 12;12:776. 10.1186/1471-2458-12-776 PMC3584983 22971152

[54] Kaess M, Brunner R, Parzer P, Carli V, Apter A, Balazs JA et al. Risk-behaviour screening for identifying adolescents with mental health problems in Europe. Eur Child Adolesc Psychiatry. 2014 Jul 1;23(7):611–20. 10.1007/s00787-013-0490-y 24248753

[55] Kaufman J, Birmaher B, Brent D, Rao U, Flynn C, Moreci P, et al. Schedule for affective Disorders and Schizophrenia for School-Age Children-Present and Lifetime Version (K-SADS-PL): initial reliability and validity data. J Am Acad Child Adolesc Psychiatry. 1997 Jul;36(7):980–8. 10.1097/00004583-199707000-00021 9204677

[56] Laurens KR, Hobbs MJ, Sunderland M, Green MJ, Mould GL. Psychotic-like experiences in a community sample of 8000 children aged 9 to 11 years: an item response theory analysis. Psychol Med. 2012 Jul;42(7):1495–506. 10.1017/S0033291711002108 21999924

[57] Beck AT, Steer RA, Brown GK (1996). Manual for the Beck Depression Inventory-II.

[58] Zung WW. A rating instrument for anxiety disorders. Psychosomatics. 1971 Dec;12(6):371–9. 10.1016/S0033-3182(71)71479-0 5172928

[59] Kelleher I, Keeley H, Corcoran P, Ramsay H, Wasserman C, Carli V, et al. Childhood trauma and psychosis in a prospective cohort study: cause, Effect, and directionality. Am J Psychiatry. 2013 Jul;1(7):734–41. 10.1176/appi.ajp.2012.12091169 23599019

[60] StataCorp StataCorp. 2015. Stata Statistical Software: Release 14. College Station, TX: StataCorp LP. College Station, TX; 2015.

[61] Healy C, Cannon M. We Need to Talk About Prevention.Am J Psychiatry. 2020 Apr1;177(4):285–7. 10.1176/appi.ajp.2020.20020155 32233678

[62] Murray RM, Cannon M (2021). Public health psychiatry: an idea whose time has come. World Psychiatry.

[63] Lustig S, Kaess M, Schnyder N, Michel C, Brunner R, Tubiana A et al. The impact of school-based screening on service use in adolescents at risk for mental health problems and risk-behaviour. Eur Child Adolesc Psychiatry [Internet]. 2022 Apr 30 [cited 2022 Oct 9]; Available from: 10.1007/s00787-022-01990-z 10.1007/s00787-022-01990-z PMC10460322 35488938

[64] Cotter P, Kaess M, Corcoran P, Parzer P, Brunner R, Keeley H, et al. Help-seeking behaviour following school-based screening for current suicidality among european adolescents. Soc Psychiatry Psychiatr Epidemiol. 2015 Jun;50(6):973–82. 10.1007/s00127-015-1016-3 25656270

[65] McCarney R, Warner J, Iliffe S, van Haselen R, Griffin M, Fisher P. The Hawthorne Effect: a randomised, controlled trial. BMC Med Res Methodol. 2007 Jul;3(1):30. 10.1186/1471-2288-7-30 PMC1936999 17608932

[66] McMahon EM, Corcoran P, Keeley H, Clarke M, Coughlan H, Wasserman D, et al. Risk and protective factors for psychotic experiences in adolescence: a population-based study. Psychol Med. 2020 Feb;6(7):1–9. 10.1017/S0033291719004136 32026792

[67] Healy C, Coughlan H, Clarke M, Kelleher I, Cannon M. What mediates the longitudinal relationship between psychotic experiences and psychopathology? J Abnorm Psychol. 2020 Jul;129(5):505–16. 10.1037/abn0000523 32309957

[68] Byrne SL, Hooke GR, Newnham EA, Page AC. The effects of progress monitoring on subsequent readmission to psychiatric care: a six-month follow-up. J Affect Disord. 2012 Mar;137(1–3):113–6. 10.1016/j.jad.2011.12.005 22244373

[69] Hasmi L, Pries LK, ten Have M, de Graaf R, van Dorsselaer S, Bak M et al. What makes the psychosis ‘clinical high risk’ state risky: psychosis itself or the co-presence of a non-psychotic disorder? Epidemiol Psychiatr Sci. 2021 Jul 6;30:e53. 10.1017/S204579602100041X PMC8264801 34225831

[70] Gronholm PC, Nye E, Michelson D. Stigma related to targeted school-based mental health interventions: A systematic review of qualitative evidence.J Affect Disord. 2018 Nov1;240:17–26. 10.1016/j.jad.2018.07.023 30041074

[71] Caldwell DM, Davies SR, Hetrick SE, Palmer JC, Caro P, López-López JA, et al. School-based interventions to prevent anxiety and depression in children and young people: a systematic review and network meta-analysis. Lancet Psychiatry. 2019 Dec;6(12):1011–20. 10.1016/S2215-0366(19)30403-1 PMC7029281 31734106

[72] Richter A, Sjunnestrand M, Romare Strandh M, Hasson H. Implementing School-Based Mental Health Services: a scoping review of the literature summarizing the factors that affect implementation. Int J Environ Res Public Health. 2022 Jan;19(6):3489. 10.3390/ijerph19063489 PMC8948726 35329175

[73] Fazel M, Hoagwood K. School mental health: integrating young people’s voices to shift the paradigm. Lancet Child Adolesc Health. 2021 Mar 1;5(3):156–7. 10.1016/S2352-4642(20)30388-6 33484659

