# Correction to: Investigating the effectiveness of three school based interventions for preventing psychotic experiences over a year period – a secondary data analysis study of a randomized control trial

**DOI:** 10.1186/s12889-023-15461-w

**Published:** 2023-03-23

**Authors:** Lorna Staines, Colm Healy, Paul Corcoran, Helen Keeley, Helen Coughlan, Elaine McMahon, Padraig Cotter, David Cotter, Ian Kelleher, Camilla Wasserman, Romuald Brunner, Michael Kaess, Marco Sarchiapone, Christina W. Hoven, Vladimir Carli, Danuta Wasserman, Mary Cannon

**Affiliations:** 1 Department of Psychiatry, Royal College of Surgeons in Ireland, 123 St Stephens Green, Dublin, Ireland; 2 National Suicide Research Foundation, Cork, Ireland; 3 School of Public Health, University College Cork, Cork, Ireland; 4 Child and Adolescent Mental Health Services North Cork, Health Service Executive, Cork, Ireland; 5 Research Society of Process Oriented Psychology United Kingdom (RSPOPUK), Old Hampstead Townhall 213 Haverstock Hill, NW3 4QP London, UK; 6 Park Royal Centre for Mental Health, Central and North West London (CNWL) NHS Trust, Central Way, Off Acton Lane, NW10 7NS London, UK; 7 Department of Psychiatry, Beaumont Hospital, Dublin 9, Ireland; 8 Division of Psychiatry, Centre for Clinical Brain Sciences, University of Edinburgh, EH10 5HF Edinburgh, UK; 9 Department of Child and Adolescent Psychiatry, Columbia University, New York State Psychiatric Institute, New York, NY USA; 10 National Centre for Suicide Research and Prevention of Mental lll-Health (NASP), Karolinska Institute, Stockholm, Sweden; 11 Clinic for Child and Adolescent Psychiatry, Psychosomatics and Psychotherapy, University of Regensburg, Regensburg, Germany; 12 University Hospital of Child and Adolescent Psychiatry and Psychotherapy, University of Bern, Bern, Switzerland; 13 Department of Child and Adolescent Psychiatry, Center for Psychosocial Medicine, University Hospital Heidelberg, Heidelberg, Germany; 14 Department of Medicine and Health Science, University of Molise, Campobasso, Italy; 15 Department of Epidemiology, Mailman School of Public Health, Columbia University, New York, USA

**Correction to: BMC Public Health (2023) 23:219**

10.1186/s12889-023-15107-x

The original publication of this article was missing the following funding acknowledgement:

- CH and MC are funded in part by the Health Research Board Investigator Lead Project (ILP-PHR-2019-009).

The original article has been updated.

