# Supplementary material for: Investigating the effectiveness of three school based interventions for preventing psychotic experiences over a year period – a secondary data analysis study of a randomized control trial
Source: BMC Public Health. 2023 Feb 1;23:219. doi: 10.1186/s12889-023-15107-x (PMC9890687; doi:10.1186/s12889-023-15107-x)
Supplement: Supplementary file 1 — Supplementary Material 1 [file 12889_2023_15107_MOESM1_ESM.docx]

**Supplementary Materials**

Supplementary materials to “Evidence that a school-based intervention is effective in preventing psychotic experiences in adolescents: data from a cluster-randomised controlled trial.”

**Table of Contents**

[**eMethods.** 2](#_Toc124350891)

[eTable 1. CONSORT 2010 checklist of information to include when reporting a randomized control trial 4](#_Toc124350892)

[eTable 2. Cut-off criteria in ProfScreen screening, Stage 1 6](#_Toc124350893)

[eTable 3. Baseline demographic and clinical characteristics of those with PE in the total sample and within each arm of the study. 7](#_Toc124350894)

[**eAnalysis 1. Complete Cases.** 8](#_Toc124350895)

[eTable 4. Dropout and partial data percentages by arm 8](#_Toc124350896)

[eTable 5. Effect sizes for each intervention arm at 3 months and 12 months on point prevalence of PEs in complete cases. 8](#_Toc124350897)

[eFigures 9](#_Toc124350898)

[eFigure 1. Beck depression inventory scores (top row) and Zung anxiety scores (bottom row) in participants with and without PE in the ProfScreen arm. 9](#_Toc124350899)

[eFigure 2. Beck depression inventory scores (top row) and Zung anxiety scores (bottom row) in participants with and without PE in the YAM arm 10](#_Toc124350900)

[eFigure 3. Beck depression inventory scores (top row) and Zung anxiety scores (bottom row) in participants with and without PE in the QPR arm. 11](#_Toc124350901)

[eFigure 4. Beck depression inventory scores (top row) and Zung anxiety scores (bottom row) in participants with and without PE in the control arm. 12](#_Toc124350902)

# **eMethods.**

**Trial Protocol.** Wasserman and colleagues have published the official trial protocol^1^ and the fidelity of the trial to its aims has also been published^2^. The SEYLE study was registered with the German clinical trial registry: DRKS00000214.

**Reference**

^1^ Wasserman, D., Carli, V., Wasserman, C., Apter, A., Balazs, J., Bobes, J., ... & Hoven, C. W. (2010). Saving and empowering young lives in Europe (SEYLE): a randomized controlled trial. BMC public health, 10(1), 1-14.

^2^ Carli, V., Wasserman, C., Wasserman, D., Sarchiapone, M., Apter, A., Balazs, J., ... & Hoven, C. W. (2013). The saving and empowering young lives in Europe (SEYLE) randomized controlled trial (RCT): methodological issues and participant characteristics. BMC public health, 13(1), 1-14.

**Additional Irish Specific Participant information.** A nationally representative catchment area was identified within each country. Within the Irish site, twenty-four schools in the south-west of Ireland were identified and approached for participation. Of these schools, 71% (n=17) participated. Participating schools were randomised into one of the four arms of the study, while accounting for school size. Informed consent was obtained from a parent of all participating students and assent was obtained from the students themselves. Within each school, students in all classes where the majority of adolescents were 14 years old were invited to participate (n=1602) and 69% (n=1112) took part in at least the first wave of the study. All participating students completed a self-report questionnaire in their classrooms, comprised of a number of established measures and several items developed for the SEYLE study^22^. The questionnaire was also administered at 3-months and 12-months after baseline, with 89% (n=993) taking part at 3-months and 86% (n=959) taking part at 12-months. The Irish site of SEYLE was the only site that incorporated questions on Psychotic Experiences into the study questionnaire^23^. This question specifically pertained to auditory hallucinations (PE) and has been shown to have strong sensitivity and specificity to all PEs. A flow diagram of the study can be found in Figure 1 (see main text).

## eTable 1. CONSORT 2010 checklist of information to include when reporting a randomized control trial

| **Section/Topic** | **Item No** | **Checklist item** | **Reported on page No** |
| --- | --- | --- | --- |
| **Title and abstract** | | | |
|  | 1a | Identification as a randomised trial in the title | 1 |
|  | 1b | Structured summary of trial design, methods, results, and conclusions (for specific guidance see CONSORT for abstracts) | 3 |
| **Introduction** | | | |
| Background and objectives | 2a | Scientific background and explanation of rationale | 5-6 |
|  | 2b | Specific objectives or hypotheses | 6 |
| **Methods** | | | |
| Trial design | 3a | Description of trial design (such as parallel, factorial) including allocation ratio | 7 |
|  | 3b | Important changes to methods after trial commencement (such as eligibility criteria), with reasons | (1) |
| Participants | 4a | Eligibility criteria for participants | (1) |
|  | 4b | Settings and locations where the data were collected | 7 |
| Interventions | 5 | The interventions for each group with sufficient details to allow replication, including how and when they were actually administered | 9, (1) |
| Outcomes | 6a | Completely defined pre-specified primary and secondary outcome measures, including how and when they were assessed | 9-10, (2) |
|  | 6b | Any changes to trial outcomes after the trial commenced, with reasons | N/A |
| Sample size | 7a | How sample size was determined | 11, (3) |
|  | 7b | When applicable, explanation of any interim analyses and stopping guidelines | N/A |
| Randomisation: |  |  |  |
| Sequence generation | 8a | Method used to generate the random allocation sequence | 11 |
|  | 8b | Type of randomisation; details of any restriction (such as blocking and block size) | 7 |
| Allocation concealment mechanism | 9 | Mechanism used to implement the random allocation sequence (such as sequentially numbered containers), describing any steps taken to conceal the sequence until interventions were assigned | 11, (1) |
| Implementation | 10 | Who generated the random allocation sequence, who enrolled participants, and who assigned participants to interventions | (1) |
| Blinding | 11a | If done, who was blinded after assignment to interventions (for example, participants, care providers, those assessing outcomes) and how | N/A |
|  | 11b | If relevant, description of the similarity of interventions | N/A |
| Statistical methods | 12a | Statistical methods used to compare groups for primary and secondary outcomes | 11-12 |
|  | 12b | Methods for additional analyses, such as subgroup analyses and adjusted analyses | 11-12 |
| **Results** | | | |
| Participant flow (a diagram is strongly recommended) | 13a | For each group, the numbers of participants who were randomly assigned, received intended treatment, and were analysed for the primary outcome | 8 |
|  | 13b | For each group, losses and exclusions after randomisation, together with reasons | 8 |
| Recruitment | 14a | Dates defining the periods of recruitment and follow-up | (2) |
|  | 14b | Why the trial ended or was stopped | 8, (2) |
| Baseline data | 15 | A table showing baseline demographic and clinical characteristics for each group |  |
| Numbers analysed | 16 | For each group, number of participants (denominator) included in each analysis and whether the analysis was by original assigned groups | 8 |
| Outcomes and estimation | 17a | For each primary and secondary outcome, results for each group, and the estimated effect size and its precision (such as 95% confidence interval) | 13-16 |
|  | 17b | For binary outcomes, presentation of both absolute and relative effect sizes is recommended | 13-17 |
| Ancillary analyses | 18 | Results of any other analyses performed, including subgroup analyses and adjusted analyses, distinguishing pre-specified from exploratory | 14-17 |
| Harms | 19 | All important harms or unintended effects in each group (for specific guidance see CONSORT for harms) | (2) |
| **Discussion** | | | |
| Limitations | 20 | Trial limitations, addressing sources of potential bias, imprecision, and, if relevant, multiplicity of analyses | 20 |
| Generalisability | 21 | Generalisability (external validity, applicability) of the trial findings | 20 |
| Interpretation | 22 | Interpretation consistent with results, balancing benefits and harms, and considering other relevant evidence | 17-19 |
| **Other information** | | |  |
| Registration | 23 | Registration number and name of trial registry | 3 |
| Protocol | 24 | Where the full trial protocol can be accessed, if available | 7 |
| Funding | 25 | Sources of funding and other support (such as supply of drugs), role of funders | 22 |

1. Wasserman D, Carli V, Wasserman C, Apter A, Balazs J, Bobes J, et al. Saving and Empowering Young Lives in Europe (SEYLE): a randomized controlled trial. BMC Public Health. 2010 Apr 13;10(1):192.

2. Wasserman D, Hoven CW, Wasserman C, Wall M, Eisenberg R, Hadlaczky G, et al. School-based suicide prevention programmes: the SEYLE cluster-randomised, controlled trial. The Lancet. 2015 Apr 18;385(9977):1536–44.

3. Wasserman C, Hoven CW, Wasserman D, Carli V, Sarchiapone M, Al-Halabí S, et al. Suicide prevention for youth--a mental health awareness program: lessons learned from the Saving and Empowering Young Lives in Europe (SEYLE) intervention study. BMC Public Health. 2012 Sep 12;12:776.

## eTable 2. Cut-off criteria in ProfScreen screening, Stage 1

| **Stage 1** | | |  |  |
| --- | --- | --- | --- | --- |
| Topics | | | Assessment | Cut-off for clinical interview |
| Risk-behaviour | | |  |  |
|  | Self-injury | | Modified version of Deliberate Self-Harm Inventory (DSHI), (lifetime) | ≥ 2 lifetime incidents of intentional self-harm |
|  | Substance abuse | |  |  |
|  |  | Tobacco | Tobacco use (lifetime measure) | Reported frequency of ≥ 2 cigarettes per day |
|  |  |  | Tobacco consumption (frequency) |  |
|  |  | Alcohol | Alcohol consumption (frequency) | Reported consuming unit of alcohol ≥2 times per week |
|  | | | Alcohol consumption (amount) | Reported consuming ≥ 3 units of alcohol per typical drinking occasion |
|  | | | Alcohol intoxication (lifetime) | Reported ≥ 3 experiences in lifetime of being clearly drunk |
|  | | | Alcohol hangover (lifetime) | Reported ≥ 3 experiences in lifetime of being hungover |
|  |  | Illegal drugs | Illicit drugs consumption (lifetime) | Reported ≥ 3 experiences in lifetime of illegal drug consumption |
|  | Sensation seeking and delinquent behaviour | | Participant reported experience (lifetime)  - Riding with someone who has been drinking  - Skateboarding or riding rollerblades in traffic and without a helmet  - Subway cart jumping, or held on the back of a moving vehicle  - Visiting known areas that are dangerous during night  - Sexual promiscuity (more than five sexual partners in life)  - Several experiences of unprotected sex | Gave a sum of ≥ 3 affirmative answers to questions |
|  | Excessive use of media | | Media exposure (frequency) | Reported spending minimum 5-6 hours per day watching television, playing games etc. |
|  | Truancy | | Truancy (frequency) | Reported ≥3 days missing school or class without permission in previous two weeks |
| Psychopathology | | |  |  |
|  | Suicidal ideation and attempts | | Paykel- Suicide Scale (PSS), calculated Paykel scale based on pupils’ self-report  Questions on previous suicide attempts | Reported suicidal thoughts or attempts in previous two weeks  Reported life-time history of suicide attempts |
|  | Depression (BDI) | | Beck Depression Inventory (BDI-II), calculated BDI score based from pupils’ self-report | Reported BDI score ≥ 14 (mild depression) |
|  | Anxiety (SAS) | | Zung Self-Rating Anxiety Scale (SAS), SAS-score calculated from pupils’ self-report | Reported SAS-score ≥ 45 (mild anxiety) |
|  | Loneliness/social relationship problems | | Loneliness (frequency) | Reported feeling lonely ‘most of the time’, or more often, within the last 12 months |
|  | Peer victimization | | Peer victimization (frequency) | Reported ≥ 5 incidents of being bullied within the last 12 months |
|  | Eating behaviour | | Calculation of body mass index (BMI) score | Reported with a BMI ≤ 16.5 (underweight) |

## eTable 3. Baseline demographic and clinical characteristics of those with PE in the total sample and within each arm of the study.

| **Characteristics** | **Total Sample** | | | **Controls** | | | **QPR** | | | **Awareness** | | | **Prof Screen** | | |
| --- | --- | --- | --- | --- | --- | --- | --- | --- | --- | --- | --- | --- | --- | --- | --- |
|  | No PE | PE | Comparison | No PE | PE | Comparison | No PE | PE | Comparison | No PE | PE | Comparison | No PE | PE | Comparison |
| Age ^a^ | 13.7  (0.6) | 13.7  (0.6) | 0.13 | 13.6  (0.5) | 13.7  (0.7) | -0.44 | 13.7  (0.5) | 13.9  (0.4) | -1.41 | 13.7  (0.6) | 13.6  (0.6) | 0.84 | 14.2  (0.9) | 13.8  (0.8) | 1.37 |
| Gender (% male)^b^ | 54.1 | 59.2 | 0.74 | 52.8 | 70.0 | 0.13 | 47.7 | 35.7 | 0.39 | 64.1 | 65.2 | 0.92 | 49.4 | 57.9 | 0.48 |
| Nationality  (% Irish)^b^ | 82.5 | 74.0 | 3.36 | 87.1 | 75.0 | 0.13 | 76.6 | 69.2 | 0.54 | 76.3 | 65.2 | 0.24 | 90.2 | 88.2 | 0.07 |
| Physical Victimisation ^c^ | 8.6  (86) | 27.6  (21) | **4.0**  (2.3-7.0) | 8.4  (30) | 40.0  (8) | **7.3**  (2.8-19.2) | 5.1  (11) | 14.3  (2) | 3.1  (0.6-15.5) | 11.8  (31) | 30.4  (7) | **3.3**  (1.2-8.5) | 8.5  (14) | 21.0  (4) | 2.9  (0.8-9.8) |
| Zung Anxiety Score^a^ | 31.0  (6.8) | 41.2  (11.1) | -**6.99** | 30.8  (6.7) | 38.4  (12.8) | **-2.52** | 30.8  (6.9) | 40.1  (8.5) | **-3.85** | 30.8  (6.8) | 40.6  (9.3) | **-3.75** | 32.0  (6.7) | 45.6  (12.0) | **-4.46** |
| Becks Depression Index score^a^ | 6.1  (6.2) | 17.4  (11.8) | **-7.99** | 6.2  (6.2) | 16.5  (11.5) | **-3.77** | 6.1  (6.8) | 10.3  (7.8) | 1.89 | 5.8  (5.8) | 19.6  (11.3) | **-5.68** | 6.1  (6.1) | 20.6  (13.6) | **-4.47** |

Note Emboldened values denote p <.05. ^a^: comparison is a t-score with unequal variance assumed. ^b^: Comparison is a Persons chi squared value. ^c^: Comparison is an odds ratio and 95% confidence interval in parentheses.

# eAnalysis 1. Complete Cases.

In eTable 4 We provide the percentages of those who dropped out of the study in each arm as well as the percentage of those with partial PE data (relative to the total number of participants and the number of participants analysed). We also reran the analysis to the main investigation using complete cases only. The interaction results are presented in the table below (eTable 5). As can be seen in the table, even when the sample is limited to complete cases, there is a significant interaction indicating a reduction in PE in the Profscreen arm of study at 12-months follow-up. This interaction is evident both before and after significant adjustment.

## **eTable 4.** **Dropout and partial data percentages by arm**

|  | **Controls** | **QPR** | **YAM** | **Profscreen** |
| --- | --- | --- | --- | --- |
| **Total Cases Analysed (%)** | 90.7 | 91.8 | 88.9 | 89.9 |
| **Partial PE Data v Total (%)** | 23.9 | 18.6 | 26.4 | 24.5 |
| **Partial PE Data v Total Analysed (%)** | 26.3 | 20.3 | 29.9 | 27.2 |
| **Complete Cases (%)** | 74.8 | 78.8 | 72.6 | 74.5 |
| **Dropped out (%)** | 10.8 | 10.4 | 14.6 | 15.4 |

## eTable 5. Effect sizes for each intervention arm at 3 months and 12 months on point prevalence of PEs in complete cases.

|  | **QPR** | | | **YAM** | | | | **ProfScreen** | | | |  |
| --- | --- | --- | --- | --- | --- | --- | --- | --- | --- | --- | --- | --- |
|  | **3-months** | **12-months** | | **3-months** | | **12-months** | | **3-months** | | **12-months** | |  |
| **Auditory Hallucinations ^a^** | | | | | |  | |  | |  | |  |
| **Univariate** | 0.93  (0.19-4.46) | | 0.36  (0.06-2.00) | | 1.04  (0.24-4.51) | | 0.91  (0.21-3.88) | | 0.63  (0.14-2.84) | | **0.15**  (0.03-0.88) | |
| **Adjustment 1.**  Baseline PEs | 0.90  (0.18-4.29) | | 0.33  (0.06-1.90) | | 1.06  (0.25-4.47) | | 0.92  (0.22-3.87) | | 0.60  (0.13-2.71) | | **0.16**  (0.03-0.86) | |
| **Adjustment 2.**  Baseline PEs and other characteristics | 0.86  (0.16-4.41) | | 0.33  (0.05-2.06) | | 1.09  (0.26-4.55) | | 1.09  (0.27-4.54) | | 0.57  (0.12-2.65) | | **0.14**  (0.02-0.85) | |

Note: ^a^ : Results are odds ratios. Other characteristic included age, gender and nationality.

# eFigures

## **eFigure 1. Beck depression inventory scores (top row) and Zung anxiety scores (bottom row) in participants with and without PE in the ProfScreen arm.**


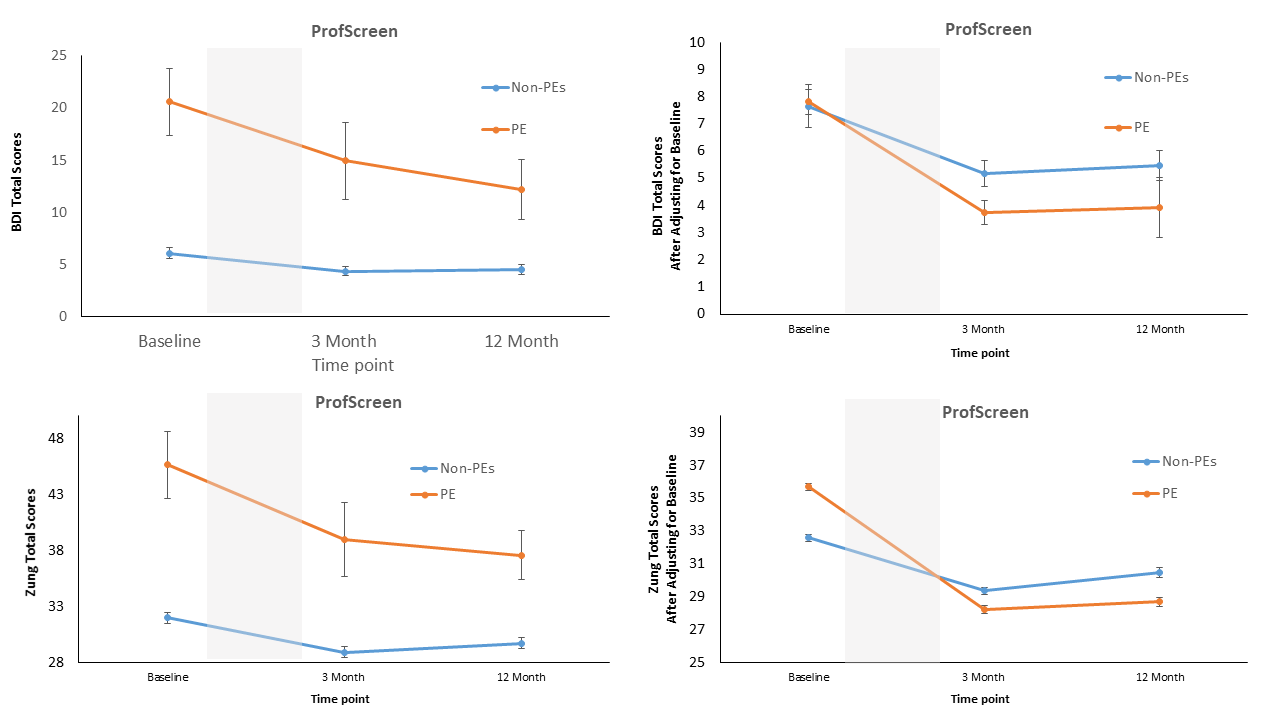


Note: Error bars represent +/-1 standard error of the mean. Graphs on the left side scores are before adjustment for baseline depression or anxiety and graphs on the right are after baseline adjustment.

## **eFigure 2. Beck depression inventory scores (top row) and Zung anxiety scores (bottom row) in participants with and without PE in the YAM arm**

Note: Error bars represent +/-1 standard error of the mean. Graphs on the left side scores are before adjustment for baseline depression or anxiety and graphs on the right are after baseline adjustment.


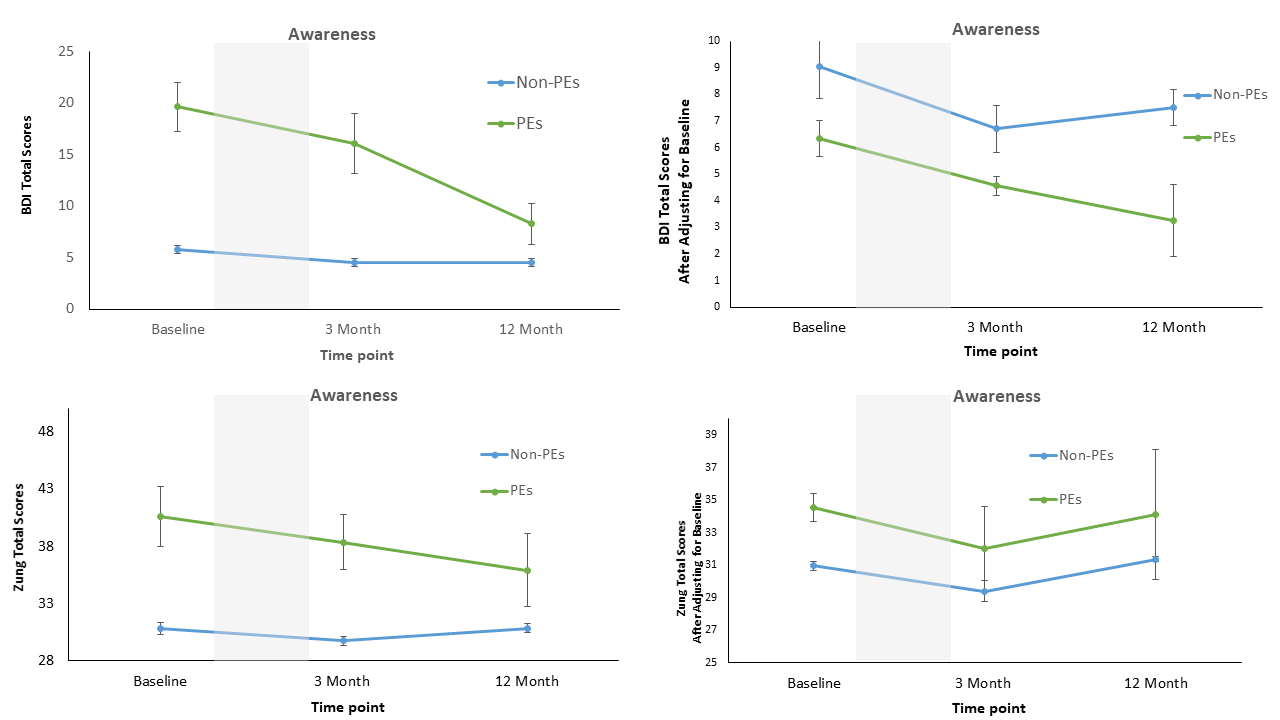


## eFigure 3. Beck depression inventory scores (top row) and Zung anxiety scores (bottom row) in participants with and without PE in the QPR arm.


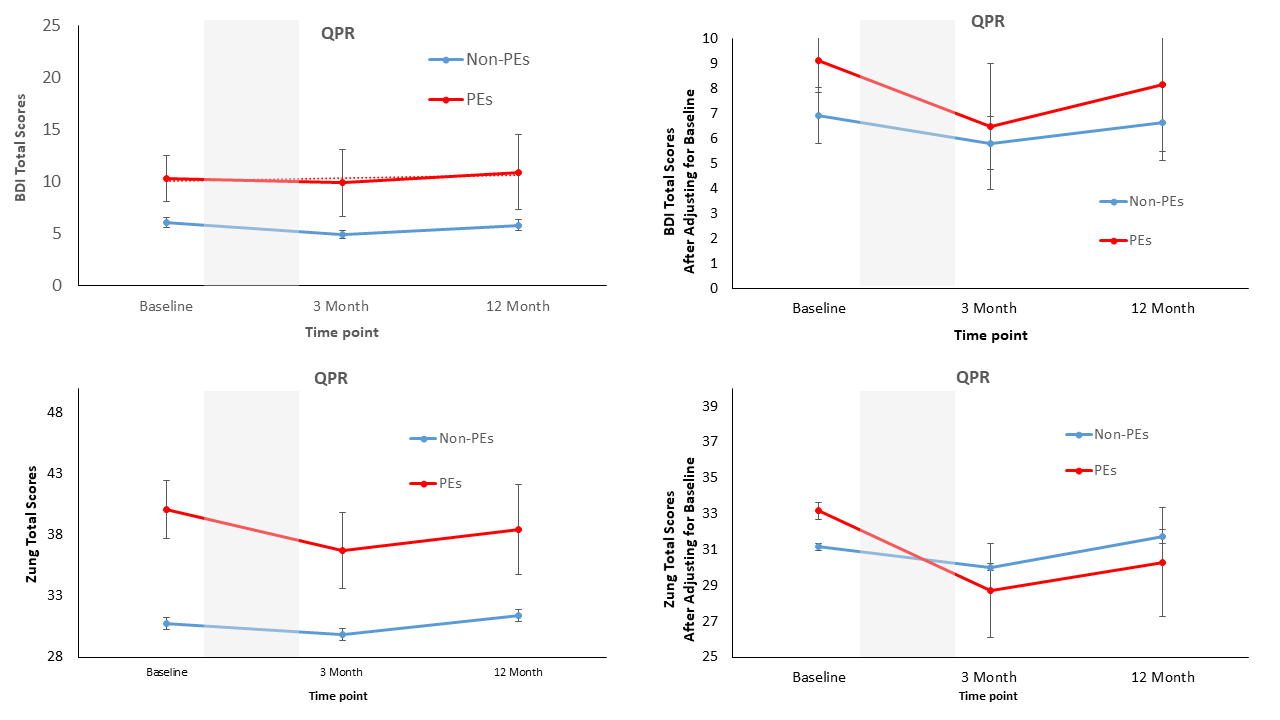


Note: Error bars represent +/-1 standard error of the mean. Graphs on the left side scores are before adjustment for baseline depression or anxiety and graphs on the right are after baseline adjustment.

## eFigure 4. Beck depression inventory scores (top row) and Zung anxiety scores (bottom row) in participants with and without PE in the control arm.


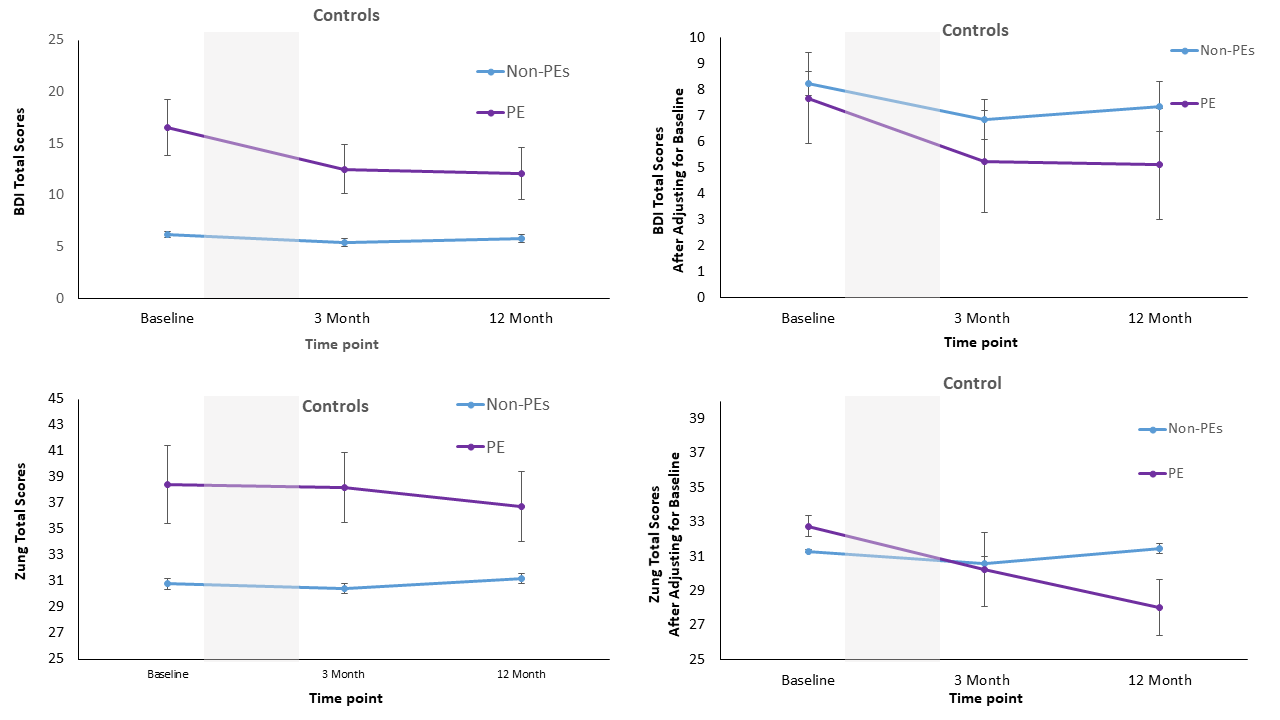


Note: Error bars represent +/-1 standard error of the mean. Graphs on the left side scores are before adjustment for baseline depression or anxiety and graphs on the right are after baseline adjustment.
